# Patients’ Expectations in Emergency Department at King Abdulaziz University Hospital: A Cross-Sectional Survey-Based Study

**DOI:** 10.7759/cureus.54211

**Published:** 2024-02-14

**Authors:** Ahmad H Bakhribah, Ghaida A. Eissa, Dania W Alsulami, Marah A Alotaibi, Htoun M Abdulmannan, Imad M Khojah

**Affiliations:** 1 Emergency Medicine, King Abdulaziz University Hospital, Jeddah, SAU; 2 Medical School, King Abdulaziz University Faculty of Medicine, Jeddah, SAU; 3 Miscellaneous, King Abdulaziz University Hospital, Jeddah, SAU; 4 Emergency Medicine, King Abdulaziz University Faculty of Medicine, Jeddah, SAU

**Keywords:** er visits, ed in saudi arabia, er expectations, emergency department, patients expectations

## Abstract

Background: Emergency departments (EDs) are vital to the timely and necessary care that a significant percentage of patients get. Emergency medicine places a high priority on quality, and to deliver patient-centered care, it is crucial to first investigate patient expectations from ER visits.

Methodology: This is a cross-sectional study of all visits to the King Abdulaziz University Hospital Emergency Department in Jeddah. The study included adult patients who visited the emergency department and were willing to complete a survey and participate in an interview. Data was collected through face-to-face questionnaires. Patient’s expectations during their visit to the emergency department were correlated to different parameters using univariate and bivariate analysis.

Results: The study included 291 patients. The majority of patients believed that their medical condition required admission to the hospital and agreed that it would be easier to receive treatment if admitted to the hospital. Results showed that 65.6% (n=191) of patients reported that the most serious patients should be seen first, and 65.3% (n=190) reported that a doctor should determine the seriousness of their health problem upon arrival. There was no significant difference found between age groups in relation to other items of expectations.

Conclusion: It is clear that a sizable percentage of patients place a high value on seeing the most urgent cases first and having a doctor assess each patient's condition when they arrive. Our results show that, in order to guarantee the best patient happiness and care, healthcare practitioners must meet patients' expectations and modify their strategies accordingly.

## Introduction

Emergency medicine is a medical specialty that diagnoses and treats traumatic and acutely ill patients. Currently, emergency departments (EDs) play a crucial role in providing timely and essential care to a substantial number of patients [1]. However, the issue of overcrowding in these departments has emerged as a significant challenge for the healthcare system. A study conducted in a tertiary hospital in Riyadh, Saudi Arabia, revealed that a total of 82,046 patients made 150,727 visits to the ED during one year [2]. Consequently, it has become imperative to explore effective strategies for meeting and managing patients' needs and expectations and reducing the number of visits to the same patient.

Since quality is considered a primary focus in emergency medicine, and to provide patient-centered care, it is essential to first explore a detailed understanding of patient expectations. Numerous studies have been conducted worldwide to evaluate the expectations within emergency departments (EDs) [3-5]. In a study conducted at the University of Calgary, they stated that expectations remained consistent regardless of triage level. The majority of patients emphasized the significance of effective communication and minimal waiting times [5].

Comprehending patient expectations is crucial in the ED. Therefore, to enhance communication between patients and healthcare workers, several studies are now prioritizing the examination of predictive factors that impact overall patient satisfaction in emergency care. Factors such as waiting times, initial assessment, and expectations from the emergency medicine visit all have a significant influence on overall patient satisfaction [6-8]. This understanding will enable healthcare providers to effectively meet the needs of each patient, thereby improving the overall effectiveness of the care provided.

This study aims to assess patients’ expectations regarding waiting hours, recurrent updates, and staff communication at King Abdulaziz University Hospital Emergency Department. It is a crucial step in understanding the current strategy and determining the best approach to achieving ED purposes and providing high-quality emergency care to each patient.

## Materials and methods

Study design

This is a cross-sectional study of all ED visits at King Abdulaziz University Hospital (KAUH) in Jeddah, Saudi Arabia. This study was conducted from August 1 to November 30, 2023.

Study area and population

The inclusion criteria for the study were patients aged > 18 who visited the ED at KAUH between August and November 2023. The exclusion criteria included patients under 18 years of age, those who did not complete the survey because either they refused or were referred to another department, and patients who were too ill or distressed. All patients who arrived at the ED were triaged using the Canadian Triage and Acuity Scale (CTAS), a five-level triage acuity scale corresponding to 1 (resuscitation), 2 (emergent), 3 (urgent), 4 (less urgent), and 5 (non-urgent). During registration, patients were assigned to their respective ED sections based on their determined CTAS level [1-5]. The researcher collected the data and interviewed the patients by themselves.

Data collection

Data were collected through face-to-face questionnaires filled out by the research team. Participants were informed about the study's objectives, methodology, potential risks, and the voluntary nature of their participation. The confidentiality of all responses was emphasized by the researcher. The questionnaire included demographic information such as age, sex, nationality, and educational level as well as chronic diseases, CTAS level, current patient status, and detailed questions about patients' expectations.

Data analysis

The data were statistically analyzed using the SPSS application v. 26 (IBM Corp., Armonk, NY) to investigate the association between the variables, the chi-squared test (χ2) was applied to qualitative data expressed as numbers and percentages. A p-value of less than 0.05 was considered statistically significant.

Ethical consideration

Ethical approval was obtained from the research ethics committee at KAUH in Jeddah, Saudi Arabia with reference number 281-23. Verbal and written consent was taken from all participants.

## Results

The study included 291 patients, of whom 46.7% (n=136) were aged between 35-64 years and 39.5% (n=115) between 18-34 years. More than half 59.1% (n=172) were females, 73.9% (n=215) were Saudi nationals, and 42.3% (n=123) had completed college or university education. Approximately 42% (n=124) had chronic diseases, with hypertension (HTN) 27.8% (n=81) and diabetes mellitus 26.55% (n=77) being the most common. The majority of patients 48.8% (n=142) were Level 3 on the Canadian Triage and Acuity Scale (CTAS) and 52.2% (n=152) of them were admitted (Table 1).

**Table 1:** Distribution of studied patients according to their demographic characteristics, chronic diseases, CTAS level, and patient status (n=291) n: total number; DM: diabetes mellitus; HTN: hypertension; CHF: congestive heart failure; MI: myocardial infarction; CVA: cerebrovascular accident: HLD: hypersensitivity lung disease; CTAS: Canadian Triage and Acuity Scale

Variable	n (%)
Age (in years)	
18-34	115 (39.5)
35-63	136 (46.7)
64 and more	40 (13.7)
Gender	
Female	172 (59.1)
Male	119 (40.9)
Nationality	
Non-Saudi	76 (26.1)
Saudi	215 (73.9)
Level of education	
Not educated	42 (14.4)
Elementary	2 (0.7)
Elementary-Middle school	30 (10.3)
High school	70 (24.1)
College or university - incomplete	24 (8.2)
College or university - complete	123 (42.3)
Chronic diseases	
DM (diabetes mellitus	77 (26.5)
HTN	81 (27.8)
CHF (congestive heart failure	20 (6.9)
MI (myocardial infarction)	14 (4.8)
CVA (Cerebrovascular accident	8 (2.7)
HLD (hypersensitivity lung disease	19 (6.5)
Renal disease	30 (10.3)
CTAS	
Levels 1-2	51 (17.5)
Level 3	142 (48.8)
Levels 4-5	98 (33.7)
Patient status	
Admitted	152 (52.2)
Discharged	88 (29.6)
Left without being seen	53 (18.2)

Regarding patients' expectations in the emergency department, Table 2 shows that 65.6% (n=191) of patients reported that the most serious patients should be seen first, and 65.3% (n=190) reported that a doctor should determine the seriousness of their health problem upon arrival. About 41.9% (n=122) mentioned that a staff member should provide them with updates every 15 minutes in the waiting room, and only 21.3% expected the update to take more than 60 minutes. Around 44% (n=128) mentioned that the reasonable waiting time for a non-life-threatening problem before being taken to the treatment room is one to two hours. The majority of patients 74.9% (n=218) believed that their medical condition required admission to the hospital. Additionally, a majority 83.1% (n=242) agreed that it would be easier to receive treatment if admitted to the hospital.

**Table 2:** Participants' expectations in the emergency department (n=291) n=total number

Variable	n (%)
Which patients should be seen first?	
If brought by ambulance	48 (16.5)
Most in pain	44 (15.1)
Most serious	191 (65.6)
Waited longest	8 (2.7)
After arriving, who should the seriousness of your health problem be determined by?	
A doctor	190 (65.3)
A triage nurse/ standard	45 (15.6)
Your own judgment	56 (19.2)
How often should staff update you in the waiting room?	
Every 15 min	122 (41.9)
Every 30 min	107 (36.8)
Every 60 min	41 (14.1)
Every 90 min	21 (7.2)
What is the reasonable time to wait with a non–life-threatening problem before being taken to the treatment room?	
<30 min	93 (32)
1-2 hrs	128 (44)
2-4 hrs	27 (9.3)
Depends on the bed status	43 (14.8)
Do you think your medical condition needed admission to the hospital?	
No	73 (25.1)
Yes	218 (74.9)
Is it easier to provide treatment for you if you are admitted to the hospital?	
Strongly agree	159 (54.6)
Agree	83 (28.5)
Neutral	27 (9.3)
Disagree	15 (5.2)
Strongly disagree	7 (2.4)

It was found that patients aged between 35 and 63 years had a significantly higher percentage of individuals who reported that the most serious patients should be seen first compared to other age groups. Additionally, 32.5% (n=13) of elderly patients aged 64 and older expected to be seen first if they were brought by ambulance (p=<0.05). Additionally, elderly patients (aged 64 or older) had a significantly higher percentage of individuals who reported that, upon arrival, a doctor should determine the seriousness of their health problem (p=<0.05). There was no significant difference found between age groups for other items of expectations (p>=0.05) (Table 3). On the other hand, there was no significant relationship found between patients' gender, nationality, or educational level and all items of patients' expectations in the emergency department (p>=0.05).

**Table 3:** Relationship between patients' age and their expectations in emergency department P-value <0.05 was considered significant. n: total number, X2: chi-square test

Variable	Age (years); n(%)			χ2	p-value
	18-34	35-63	64 and more		
Which patients should be seen first?				13.73	0.033
If brought by ambulance	19 (16.5)	16 (11.8)	13 (32.5)		
Most in pain	15 (13)	26 (19.1)	3 (7.5)		
Most serious	76 (66.1)	92 (67.6)	23 (57.5)		
Waited longest	5 (4.3)	2 (1.5)	1 (2.5)		
After arriving, should the seriousness of your health problem be determined by?				11.11	0.025
A doctor	68 (67.8)	83 (61)	29 (72.9)		
A triage nurse/ standard	11 (9.6)	31 (22.8)	3 (7.5)		
Your own judgment	26 (22.6)	22 (16.2)	8 (20)		
How often should staff update you in the waiting room?				1.26	0.974
Every 15 min	46 (40)	57 (41.9)	19 (47.5)		
Every 30 min	42 (36.5)	51 (37.5)	14 (35)		
Every 60 min	18 (15.7)	19 (14)	4 (10)		
Every 90 min	9 (7.8)	9 (6.6)	3 (7.5)		
What is the reasonable time to wait with a non–life-threatening problem before being taken to the treatment room?				8.09	0.231
<30 min	29 (25.2)	53 (39)	11 (27.5)		
1-2 hrs	53 (46.1)	57 (41.9)	18 (45)		
2-4 hrs	12 (10.4)	12 (8.8)	3 (7.5)		
Depends on the bed status	21 (18.3)	14 (10.3)	8 (20)		
Do you think your medical condition needed admission to the hospital?				1.85	0.395
No	26 (22.6)	39 (28.7)	8 (20)		
Yes	89 (77.4)	97 (71.3)	32 (80)		
Is it easier to provide treatment for you if you were admitted to the hospital?				10.76	0.215
Strongly disagree	2 (1.7)	5 (3.7)	0 (0.0)		
Disagree	6 (5.2)	6 (4.4)	3 (7.5)		
Neutral	17 (14.8)	8 (5.9)	2 (5)		
Agree	35 (30.4)	37 (27.2)	11 (27.5)		
Strongly agree	55 (47.8)	80 (58.8)	24 (60)		

As shown in Tables 4-5, patients with chronic diseases and those with CTAS Levels 1-2 had a significantly higher percentage of patients who believed that their medical condition required admission to the hospital (p=<0.05).

**Table 4:** Relationship between having chronic diseases and patients' expectations in the emergency department P-value <0.05 was considered significant n: total number; X2: chi-square test

Variable	Do you have any chronic diseases?	
	Yes; n (%)	No; n (%)
Which patients should be seen first?		
If brought by ambulance	25 (20.2)	23 (13.8)
Most in pain	22 (17.7)	22 (13.2)
Most serious	74 (59.7)	117 (70.1)
Waited longest	3 (2.4)	5 (3)
After arriving, who should the seriousness of your health problem be determined by?		
A doctor	74 (59.7)	116 (69.5)
A triage nurse/ standard	19 (15.3)	26 (15.6)
Your own judgment	31 (25)	25 (15)
How often should staff update you in the waiting room?		
Every 15 min	50 (40.3)	72 (43.1)
Every 30 min	47 (37.9)	60 (35.9)
Every 60 min	20 (16.1)	21 (12.6)
Every 90 min	7 (5.6)	14 (8.4)
What is the reasonable time to wait with a non–life-threatening problem before being taken to the treatment room?		
<30 min	36 (29)	57 (34.1)
1-2 hrs	57 (46)	71 (42.5)
2-4 hrs	11 (8.9)	16 (9.6)
Depends on the bed status	20 (16.1)	23 (13.8)
Do you think your medical condition needed admission to the hospital?		
No	21 (16.89)	52 (31.1)
Yes	103 (83.1)	115 (68 .9)
Is it easier to provide treatment for you if you were admitted to the hospital?		
Strongly disagree	3 (2.4)	4 (2.4)
Disagree	5 (4)	10 (6)
Neutral	12 (9.7)	15 (9)
Agree	41 (33.1)	42 (25.1)
Strongly agree	63 (50.8)	96 (57.6)

**Table 5:** Relationship between CTAS and patients' expectations in the emergency department P-value <0.05 was considered significant n: Total number, X2: chi-square test; CTAS: Canadian Triage and Acuity Scale

Variable	CTAS		
	Levels 1-2; n(%)	Level 3; n(%)	Levels 4-5; n(%)
Which patients should be seen first?			
If brought by ambulance	10 (19.6)	23 (16.2)	15 (15.3)
Most in pain	6 (11.8)	27 (19)	11 (11.2)
Most serious	34 (66.7)	89 (62.7)	68 (69.4)
Waited longest	1 (2)	3 (2.1)	4 (4.1)
After arriving, who should the seriousness of your health problem be determined by?			
A doctor	35 (68.6)	91 (64.1)	64 (65.3)
A triage nurse/ standard	5 (9.8)	19 (13.4)	21 (21.4)
Your own judgment	11 (21.6)	32 (22.5)	13 (13.3)
How often should staff update you in the waiting room?			
Every 15 min	21 (41.2)	57 (40.1)	44 (44.9)
Every 30 min	20 (39.2)	54 (38)	33 (33.7)
Every 60 min	6 (11.8)	18 (12.7)	17 (17.3)
Every 90 min	4 (7.8)	13 (9.2)	4 (4.1)
What is the reasonable time to wait with a non–life-threatening problem before being taken to the treatment room?			
<30 min	17 (33.3)	40 (28.2)	36 (36.7)
1-2 hrs	25 (49)	59 (41.5)	44 (44.9)
2-4 hrs	1 (2)	16 (11.3)	10 (10.2)
Depends on the bed status	8 (15.7)	27 (19)	8 (8.2)
Do you think your medical condition needed admission to the hospital?			
No	7 (13.7)	30 (21.1)	36 (36.7)
Yes	44 (86.3)	112 (78.9)	62 (63.3)
Is it easier to provide treatment for you if you were admitted to the hospital?			
Strongly disagree	0 (0.0)	6 (4.2)	1 (1)
Disagree	2 (3.9)	5 (3.5)	8 (8.2)
Neutral	5 (9.8)	13 (9.2)	9 (9.2)
Agree	15 (29.4)	42 (29.6)	26 (26.5)
Strongly agree	29 (56.9)	76 (53.5)	54 (55.1)

## Discussion

Our study aimed to measure patients' expectations in the ED and their relation to gender, age, and educational level. To improve the quality of care, we conducted this study on 291 patients who visited our academic tertiary hospital in Saudi Arabia. The study can be a road map for tertiary hospitals to improve the patient's experience based on their expectations. 

The admission rate from the ED is approximately 20% worldwide [9]. In our research, we found that 52.2% (n=152) of patients were admitted to the hospital, whereas 48.8% (n=142) were categorized as level 3 CTAS. These results suggest that tertiary hospitals receive patients with more severe conditions who require hospitalization. Resulting in longer wait times for patients in the ED and negatively impacting their experience in the hospital.

Regarding patients' expectations in the ED, a significant proportion of 65.6% (n=191) reported that priority should be given to the most critically ill individuals. This statistic marks an encouraging improvement compared to a prior study wherein it was dishearteningly discovered that nearly half of the participants anticipated being attended to by healthcare providers solely based on their time of arrival, without considering the urgent or critical nature of their case [10]. Still, these findings underscore the imperative for enhanced community education and awareness initiatives aimed at bolstering patient comprehension and expectations.

In the current study, the majority 65.3% (n=190), believe that upon arriving, a doctor should determine the seriousness of the medical condition, and 41.9% (n=122) agreed that a staff member should update them every 15 minutes in the waiting room. Similarly, many previous studies agreed that more than half of the patients (76%) felt that ED staff should update patients every 30 minutes or less [10]. These studies also indicated that nearly two-thirds (62%) of the patients would like to be updated and informed about the reasons for any delay in seeing a doctor (87.5%) on at least an hourly basis (61.1%), regardless of who gives the update [5]. Additionally, nearly 75% of patients emphasized the importance of receiving periodic updates about waiting time, with 69% preferring updates every 30 minutes [11].

It was found that 44% (n=128) of the patients in our study agreed that the reasonable wait time for non-life-threatening problems before being taken to the treatment room is 1-2 hours. Moreover, 74.9% (N=218) of the patients believed that their medical condition required admission to the hospital. This could be because it is easier to arrange advanced imaging and receive proper treatment for admitted patients, which most of the participants agreed with when they were asked if it is easier to provide treatment for them if they were admitted to the hospital. Therefore, early decision-making by the emergency physician will improve patient satisfaction, which is supported by a study that showed that patients whose hospital stay was planned were psychologically and socially better prepared than those whose hospital stay was unplanned [12]. According to the findings of previous studies, informing patients about their care management plans, considering their preferences and demands, and demonstrating an understanding of their concerns may improve patients' overall satisfaction [12].

Furthermore, we found no significant relationship between patients' gender, nationality, and educational level with regard to their expectations in the ED. Similarly, a previous study also found no associations between patients’ sex, level of education, marital status, and their knowledge and anticipations of the triage system in the ED [11].

This study has limitations in terms of its generalizability to other healthcare settings, as it was conducted at a single tertiary center. The data collection process involved approaching patients in the emergency department during different shifts and on various days, which may have influenced their expectations and opinions. Patients approached during busier times might have been less able to provide accurate responses, while those approached during quieter times may have had a different perspective. The variability in shifts and days also introduces bias, making it challenging to draw definitive conclusions or generalize the findings. Further research involving multiple healthcare centers and standardized data collection procedures would enhance our understanding of patient perspectives in the emergency department.

## Conclusions

In conclusion, this study provides valuable insights into the expectations of patients in the emergency department. It is evident that a significant portion of patients prioritize the importance of seeing the most critical patients first and having a doctor determine the severity of their health problem upon arrival. Furthermore, patients between the ages of 35 and 64 years exhibited distinct expectations regarding priority and determination of severity. Most patients want to be updated about the waiting time at least every 30 minutes. These findings highlight the need for healthcare providers to address patients' expectations and tailor their approach accordingly to ensure optimal patient satisfaction and care.
